# Precision isolation and cultivation of single cells by vortex and flat-top laser ejection

**DOI:** 10.3389/fmicb.2024.1369506

**Published:** 2024-04-10

**Authors:** Fuyuan Chen, Kunxiang Liu, Lindong Shang, Yuntong Wang, Xusheng Tang, Peng Liang, Bei Li

**Affiliations:** ^1^Key Laboratory of Optical System Advanced Manufacturing Technology, Changchun Institute of Optics, Fine Mechanics and Physics, Chinese Academy of Sciences, Changchun, China; ^2^University of Chinese Academy of Sciences, Beijing, China

**Keywords:** laser induced forward transfer, vortex beam, flat-top beam, single cell sorting, single-cell culture

## Abstract

Single-cell isolation stands as a critical step in single-cell studies, and single-cell ejection technology based on laser induced forward transfer technology (LIFT) is considered one of the most promising methods in this regard for its ability of visible isolating single cell from complex samples. In this study, we improve the LIFT technology and introduce optical vortex laser-induced forward transfer (OV-LIFT) and flat-top laser-induced forward transfer (FT-LIFT) by utilizing spatial light modulator (SLM), aiming to enhance the precision of single-cell sorting and the cell’s viability after ejection. Experimental results demonstrate that applying vortex and flat-top beams during the sorting and collection process enables precise retrieval of single cells within diameter ranges of 50 μm and 100 μm, respectively. The recovery rates of *Saccharomyces cerevisiae* and *Escherichia coli DH5α* single cell ejected by vortex beam are 89 and 78%, by flat-top beam are 85 and 57%. When employing Gaussian beam sorting, the receiving range extends to 400 μm, with cultivation success rates of *S. cerevisiae* and *E. coli DH5α* single cell are 48 and 19%, respectively. This marks the first application of different mode beams in the ejection and cultivation of single cells, providing a novel and effective approach for the precise isolation and improving the viability of single cells.

## 1 Introduction

Cells, as the fundamental units of life, exert profound influence on the fields of biology and medicine through their minute structural and functional differences (Schubert, 2011; Wikswo, 2014). Isolation and cultivation of single cells allows researchers to gain insights into individual microbial characteristics, metabolic pathways, gene expression and functional diversity, especially for the as-yet-unculturable microorganisms in nature (Singer et al., 2017). It not only enriches our understanding of the microbial world, but also provides a useful means for more in-depth and comprehensive excavation of the biological properties of microorganisms (Jing et al., 2018; Potter, 2018). However, the precision of single-cell sorting and the viability and cultivation capacity of cells post-sorting remain major challenges limiting their development. Conventional methods of single-cell isolation, such as micromanipulation (Piatkevich et al., 2018), fluorescence-activated cell sorting (FACS) (Gross et al., 2015; Hu et al., 2016), laser microdissection (LMD) (Decarlo et al., 2011), and optical trapping (Ashkin et al., 1986). Micromanipulation uses microtools to separate cells under a microscope, which has a relatively low throughput and requires highly skilled specialized training (Zeb et al., 2019). FACS utilizes fluorescent markers on cell surfaces for sorting, offering high throughput and wide applicability (Adan et al., 2017). However, it is a challenge to use FACS to reveal spatial distribution of microbes and directly analyze complex samples *in situ* (Cho et al., 2010). LCM uses a focused laser to cut a cell from its surroundings, which is normally used for fixed tissue (Espina et al., 2006). Optical tweezers, on the other hand, use a laser beam to apply force to individual cells for separation (Zhong et al., 2013); however, the throughput of optical tweezers is generally low (Huang et al., 2009).

Laser-induced forward transfer (LIFT), based on the interaction principles between laser and materials (Serra and Piqué, 2019), possesses the capability to precisely transfer minute substances and has been applied in efficient isolation and cultivation of single cells (Kattamis et al., 2007; Marquez et al., 2020). Compared to other sorting techniques, LIFT cell sorting technology has a higher microscopic resolution, and can be used to sort target microbial cells based on a variety of characteristics such as morphology, size, fluorescence, and Raman molecular fingerprints (Song et al., 2017; Gan et al., 2021). Additionally, due to its capability to transfer cells from both liquid and solid samples, LIFT sorting technology enables single-cell isolation in a state closer to their original environment, making it more suitable for the separation of microorganisms in complex environments (Ringeisen et al., 2015). Haider et al. (2017) explored the impact of laser energy on the viability of *yeast* and *Escherichia coli*, utilizing titanium dioxide as the energy absorption layer. Liang et al. (2022) introduced a three-layer LIFT system designed to isolate microorganisms from the influences of heat and force, aiming to enhance microbial activity. However, the accuracy of single-cell isolation and the post-sorting vitality in LIFT still require further refinement. The current LIFT uses a Gaussian beam as a light source with a Gaussian distribution of energy, which can cause lateral thrust on single cells, affecting the accuracy of reception. And the cells will still be damaged by heat and force, affecting the viability after sorting.

In the modern field of optics, beam shaping technology is gradually becoming a forefront area of great interest (Barbotin et al., 2019; Chen et al., 2019). The technology aims to alter the traditional shape of beams, creating optical fields with unique properties and functions, thereby bringing forth exciting opportunities and challenges in the realms of optics and photonics (Mathis et al., 2012; Salter and Booth, 2019). Among these, flat-top beam has emerged as a distinctive beam shape that garners widespread interest and research, due to its flat and uniform light intensity distribution, holding significance in laser processing, imaging, optical communication, and biomedical applications (Zheng et al., 2021). Simultaneously, optical vortices, with their associated orbital angular momentum (OAM) and dark cores related to their helical wavefronts, find extensive applications in optical manipulation, high-capacity optical communication, and super-resolution imaging (Bernet et al., 2006; Jeffries et al., 2007). Flat-top beam and Vortex beam have been widely employed in LIFT for printing various materials, with their unique characteristics transcending those of traditional Gaussian beams, resulting in peculiar interactions between laser and materials (Rapp et al., 2015; Takahashi et al., 2016; Nakamura et al., 2019; Kawaguchi et al., 2022).

**Figure 1:**
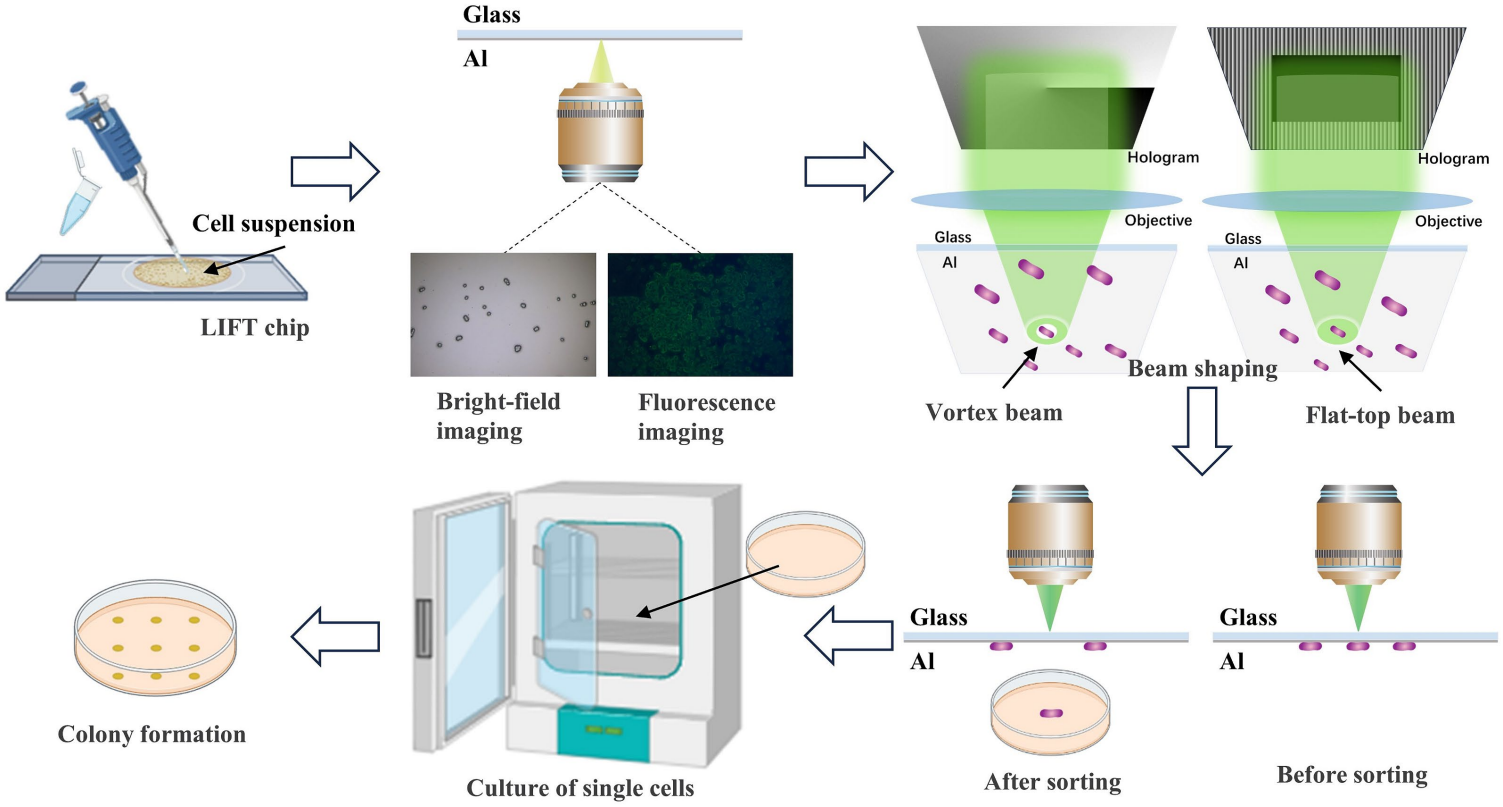
Flow diagram of sample preparation and the different mode beams ejection sorting system. Initially, the cell suspension is transferred to the sorting chip. Single cells are identified through bright-field and fluorescence imaging. Subsequently, laser beams are shaped using beam shaping techniques into vortex and flat-top beams. The laser precisely targets the absorption layer above the target single cell, executing accurate ejection sorting. Finally, the ejected single cells are collected and cultured to form colonies.

In this study, to precisely sort individual cells and enhance the post-sorting cell vitality, we innovatively employed an SLM to shape a Gaussian beam into vortex and flat-top beams. These modulations were applied in the OV-LIFT and FT-LIFT systems for ejecting single cells and subsequent cultivation, as depicted in Figure 1. The system with a circular vortex beam exerts a force converging towards the center of the cell, while the system with uniformly distributed flat-top beam exerts a downward force, enabling precise separation and collection of individual cells. The heat and force generated by the vortex beam is applied to the periphery of the cell rather than the cell itself, whereas the force generated by the flat-top beam is very uniform, so the cell suffers very little damage. Successful achievement of precise sorting and reception at the single-cell level, significantly improving the success rate of cell cultivation.

## 2 Materials and methods

### 2.1 Experimental set-up

**Figure 2:**
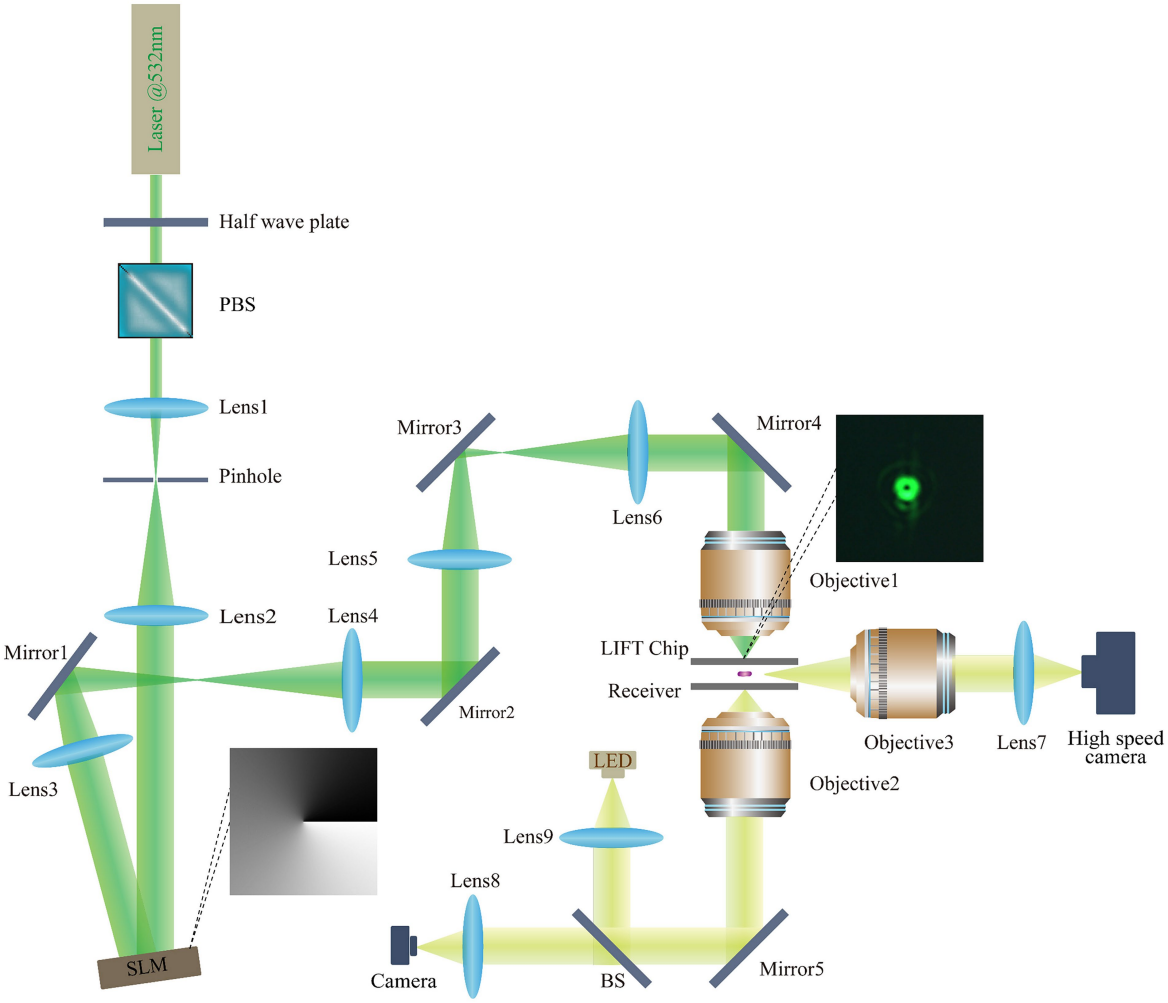
Light path diagram of the different mode modulated LIFT experimental set-up. Laser: MPL-N-532 (Changchun New Industries Optoelectronics Technology Co., Ltd.); SLM: EXULUS-HD2 (Thorlabs); Objective1: 10×/NA0.4 U Plan X Apo (Nikon); Objective2: 50×/NA0.6 TU Plan ELWD (Nikon); Objective3: 50× Plan Apo (Mitutoyo); Lens 1: *f* = 15 mm, Lens 2: *f* = 80 mm, Lens 3–6, *f* = 150 mm Lens 7–8: *f* = 125 mm, Lens 9, ACL25416U-A (Thorlabs); Mirrors 1–5: PF10-03-P01 (Thorlabs); Half-wave plate: WPHSM05-532 (Thorlabs); Polarizing beam splitter: PBS251 (Thorlabs); Beam splitter: BSW10R (Thorlabs); LED: MNWHD2 (Thorlabs); CMOS camera: U3P630-H (Shenzhen DO3THINK Technology Co., Ltd.); Fluorescent Cube: MDF-GFP2 (Thorlabs); High speed camera: AcutEye-2M-2000 (Ketianjian Photoelectricity Co., Ltd.).

The different mode modulated LIFT experimental setup is illustrated in Figure 2. A 532-nanometer single-pulse laser with a pulse duration of 2 nanoseconds (FWHM) was chosen as the light source. The energy modulation module consists of a half-wave plate (HP) and a polarizing beam splitter (PBS). To expand the laser beam, laser expansion mirrors (Lens 1, Lens 2) were employed, and the influence of stray light was minimized through pinhole filtering. The pure-phase reflective liquid-crystal-on-silicon spatial light modulator (LCOS-SLM) has a resolution of 1,920 × 1,080, operates in the wavelength range of 400–850 nanometers, and provides at least 2π phase delay for the 532-nanometer wavelength laser. Lens 3–6 constitute two 4F systems, where mirrors (mirrors 1–5) adjust the laser’s direction, and the modulated beam passes through two sets of 4F systems into the ejection objective. Two 4F systems were designed to meet spatial requirements and minimize optical intensity loss. The bottom imaging system captures cell images and is composed of a microscopic objective and a CMOS camera. During fluorescence imaging, the BS is switched to a fluorescence cube. The high-speed imaging system, comprised of a long-focal-length objective and a high-speed camera, ensures high temporal and spatial resolution, to record the whole process of microbial cells ejection.

### 2.2 Algorithm for shaping different mode beams

The algorithm for generating vortex beams employs a fork grating method. By calculating the laser wavelength, the phase of the fork-shaped grating is obtained and then loaded onto the SLM to generate the vortex beam. Adjusting the order of the grating allows the generation of vortex beams with different diameters (Haotong et al., 2013; Chen et al., 2016). The generation of flat-top beams utilizes a hybrid holographic method, combining binary gratings and geometric masks (Liu et al., 2018). This method loads a hybrid holographic pattern onto the SLM, achieving the shaping of high-quality flat-top beam. The horizontal polarization laser beam, reflected by the spatial light modulator, diffracts into multiple beams, with energy mainly distributed in the ±1 orders and 0 order. These ±1 order beams are blocked by a slit, significantly reducing diffraction effects around the shaped beam. By loading corresponding holographic patterns onto the spatial light modulator, we can conveniently obtain flat-top beam profiles of any shape.

### 2.3 Accurate single-cell ejection and reception

**Figure 3:**
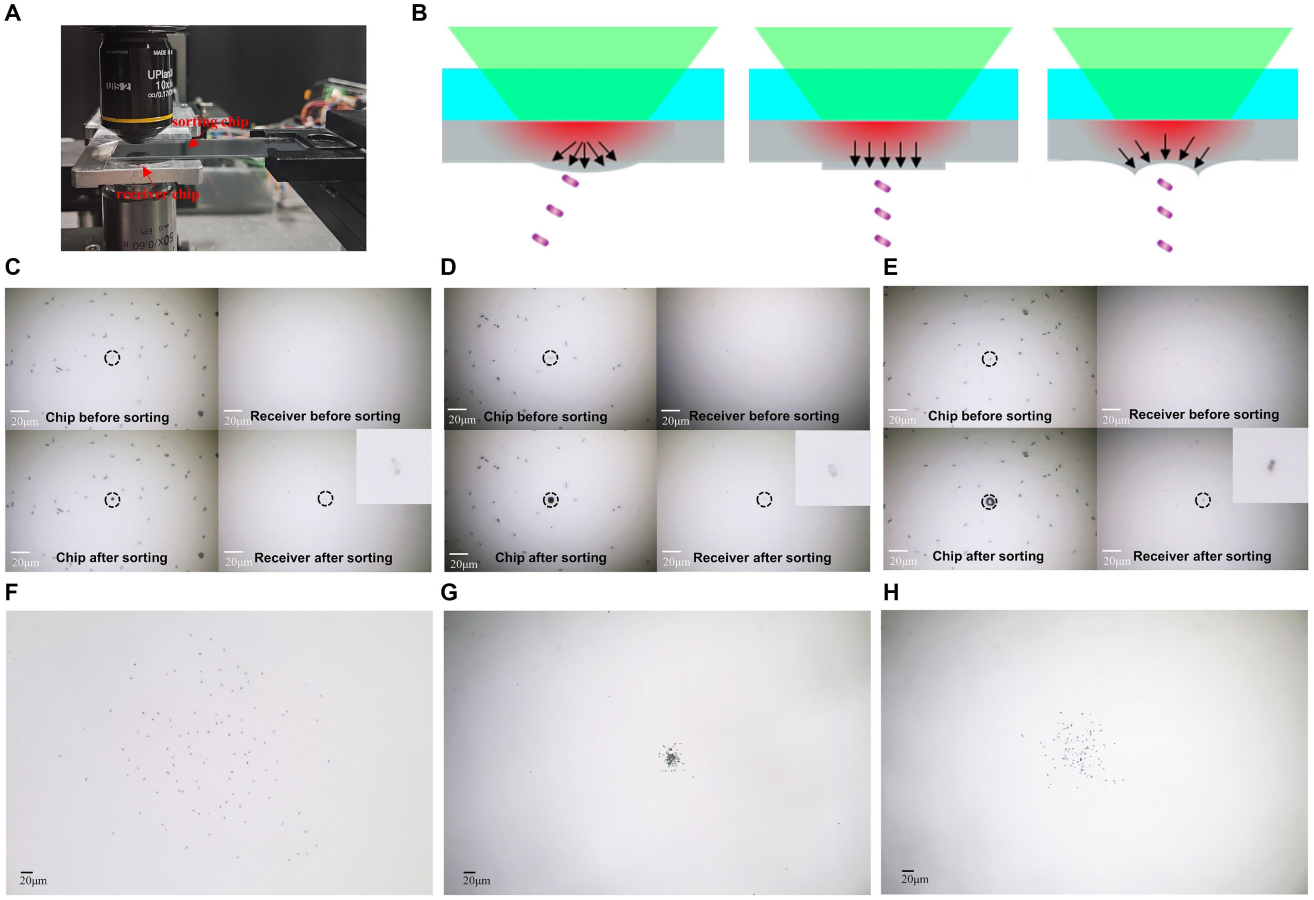
Precise ejection and reception of single cells. **(A)** Experimental setup for ejection and reception. **(B)** Schematic illustrating the influence of different mode beams on separation accuracy. **(C)** One single cell’s ejection and reception by gaussian beam. **(D)** One single cell’s ejection and reception by flat-top beam. **(E)** One single cell’s ejection and reception by vortex beam. **(F)** One hundred single cell’s ejection and reception by Gaussian beam. **(G)** One hundred single cells’ ejection and reception by flat-top beam. **(H)** One hundred single cells’ ejection and reception by vortex beam. The bar represents 20 μm.

The LIFT chip is coated with a 25-nanometer-thick aluminum film as an absorption layer. Under the action of laser pulses, the aluminum film is heated, generating powerful ejection force to achieve precise cell sorting. We directly attached the *E. coli* samples onto the LFIT chip, and then turned over the chip and mounted it on an XY displacement stage. And then observed and selected the samples under a microscope. A 0.17 mm thick cover glass was employed as the receiver to show the distribution of received cells (as shown in Figure 3A). By adjusting the imaging objective to focus on different planes, we could clearly observe the distribution of cells on both the chip and the receiver, with a distance of approximately 0.5 mm between them.

### 2.4 High-speed imaging

**Figure 4:**
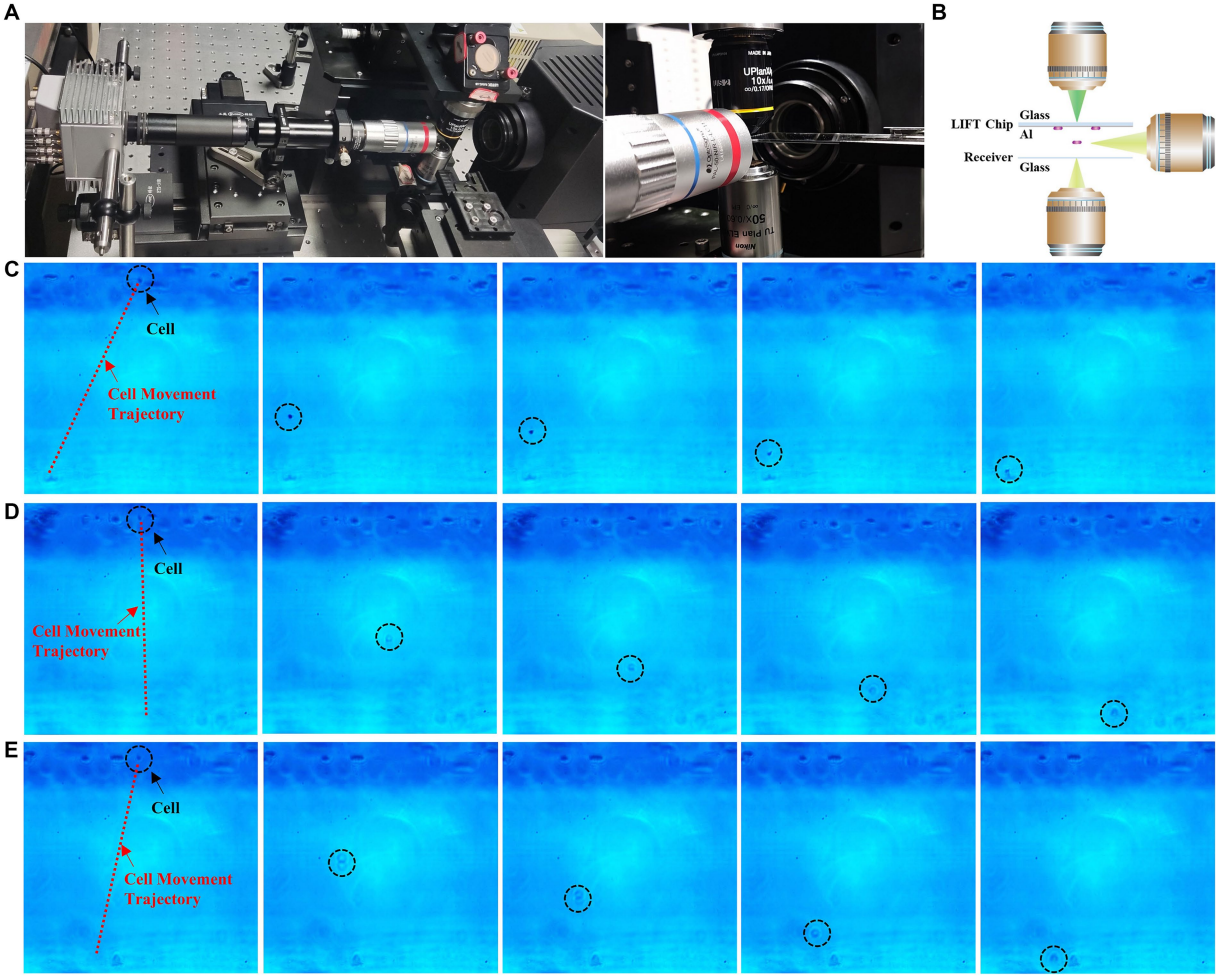
High-speed camera capture of sorting process. **(A)** High-speed camera setup. **(B)** Schematic diagram of high-speed camera unit. **(C)** Time-lapse images of Gaussian beam ejection of single cells (see Supplementary Video S1). **(D)** Time-lapse images of flat-top beam ejection of single cells (see Supplementary Video S2). **(E)** Time-lapse images of vortex beam ejection of single cells (see Supplementary Video S3).

To provide a clearer characterization of the ejection process of cells under different mode beam conditions, we established a side-view high speed microscopy imaging system, to observe the entire process of microbial cells from ejection to reception from a lateral perspective (as shown in Figure 4A). The side-view imaging objective was mounted on a XYZ three-dimensional displacement stage. By adjusting the displacement stage, the objective could be aligned to the targeted cell. The optical path behind the objective guided the image to the high-speed camera, which owns 1,920 × 1,080 pixels and the frame rate of up to 1,088 fps.

### 2.5 Single-cell sorting and cultivation

In the process of LIFT single-cell sorting and cultivation, we implemented a series of steps to ensure the accuracy and recovery rate. Initially, to ensure uniform spreading of the bacterial suspension on the aluminum film during LIFT, three layers of phytic acid PA-Al^3+^ film were attached to provide hydrophilicity (Wang et al., 2018). Precise control of the XY electrical displacement stage allowed us to select the target cells and position them at the location of the laser pulse. Pulsed laser energy used for sorting is 2.55 μJ. To prevent contamination, the entire experimental setup, chip, and receiver were placed in a cleanroom and subjected to 20 min of UV irradiation before the experiment. For the receiver, a 35 mm culture dish with agar medium was used, and the distance between the receiver and the LIFT chip was approximately 2 mm. We developed a program to control the displacement stage to stop at 9 receiving place on the culture dish. While observing bacteria or cells on the LIFT chip, the receiver was moved out of the optical path. Once the target cells were identified, the receiver was moved to the correct position to capture the ejected cells. The singly sorted cells were then cultured in the agar dish: for *Saccharomyces cerevisiae*, the dish was cultured at 30°C for 36 h, and for *E. coli*, it was cultured at 37°C for 24 h.

## 3 Results

### 3.1 Generation of different mode beams through beam shaping

**Figure 5:**
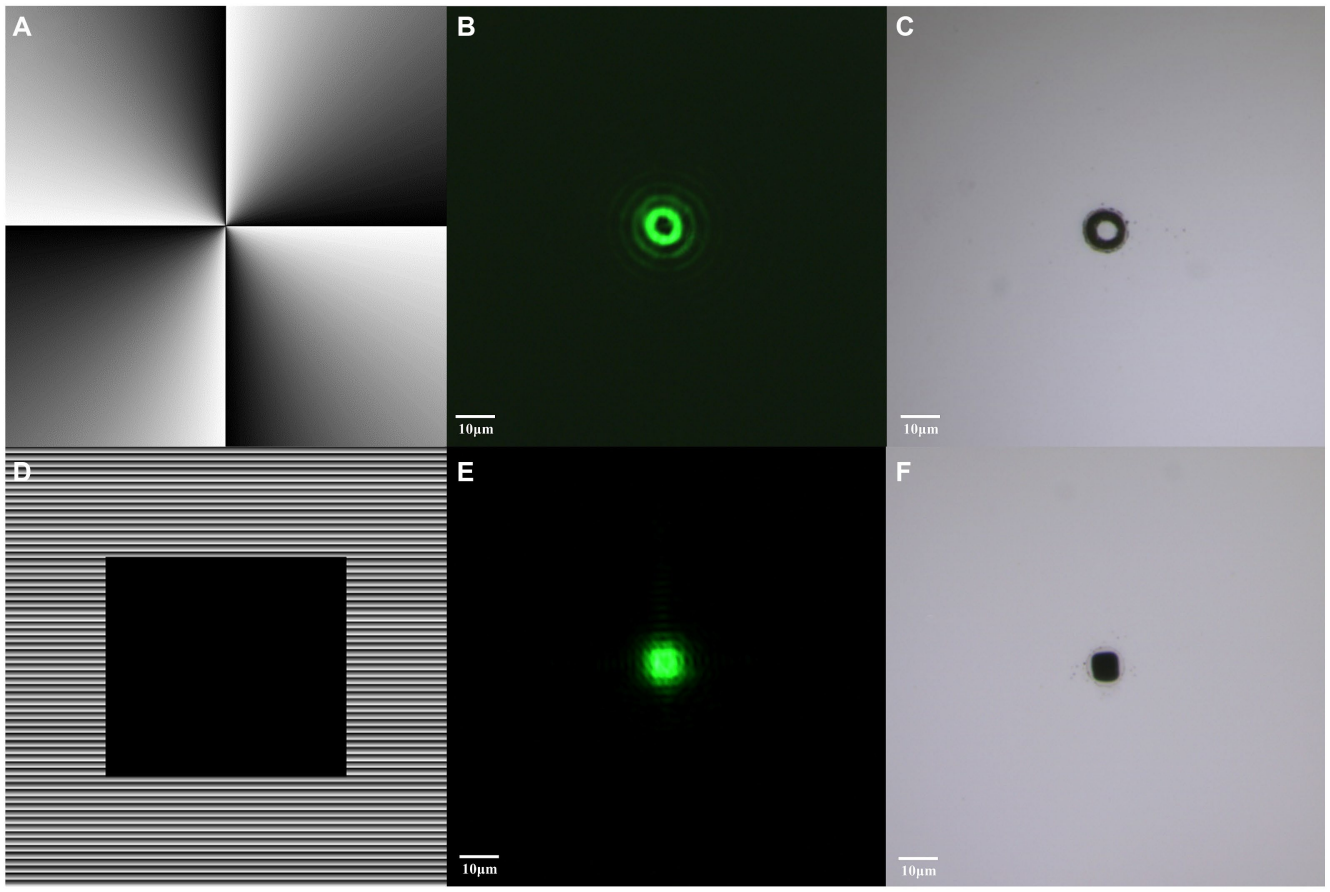
Generation of vortex and flat-top beams through beam shaping. **(A)** Hologram corresponding to vortex beam. **(B)** Distribution of modulated vortex beam. **(C)** Material transferred induced by vortex beam. **(D)** Hologram corresponding to flat-top beam. **(E)** Distribution of modulated flat-top beam. **(F)** Material transferred induced by flat-top beam. The bar represents 10 μm.

Figure 5 illustrates the process of generating different mode beams. Initially, by calculating the phase of the vortex beam at a wavelength of 532 nm and loading the phase map (Figure 5A) onto the SLM, we successfully realized the formation of vortex beam (Figure 5B). Subsequently, we employed a hybrid holographic approach for shaping the flat-top beam. Setting a geometric mask with the desired shape as zero grayscale level, creating zero-phase delay to act as a reflective mirror, and loading the hybrid phase map (Figure 5D) onto the SLM, we achieved the configuration of flat-top beams (Figure 5E).

Under the influence of pulsed laser, these two beam modes exhibited different material transfer effects, providing ideal optical tools for subsequent experiments. The vortex beam induced a circular material transfer (Figure 5C), while the flat-top beam presented a square material transfer (Figure 5F).

### 3.2 Precise isolation and reception of single cells

The precise separation and reception of single cells represent crucial steps in studying and experimenting at the single-cell level, holding paramount significance for the isolation and cultivation of individual cells. In this study, we utilized *E. coli* as a model organism for separation and reception experiments. The results demonstrate that individual *E. coli* cells can be successfully ejected from the chip by different mode beams, leaving distinct markings and effectively recovering on the receiver (Figures 3C–E). In comparison to Gaussian beam, where energy follows a Gaussian distribution, the system with a circular vortex light spot exerts a force converging towards the center of the cell, while the system with uniformly distributed flat-top beam exerts a downward force, enabling precise separation and collection of individual cells (Figure 3B). To verify the accuracy of the receiving by different modes light beam, 100 single *E. coli* cells were separately ejected by using GS-LIFT, OV-LIFT, and FT-LIFT. Cells ejected by GS-LIFT showed a distribution diameter of approximately 400 micrometers on the receiver (Figure 3F), while cells ejected by FT-LIFT exhibited a distribution diameter below 50 micrometers (Figure 3G), and OV-LIFT resulted in a distribution diameter below 100 micrometers (Figure 3H). The standardized deviation of cells ejected from FT-LIFT and OV-LIFT was 8.4 and 36.4, respectively. This indicates that the FT-LIFT and OV-LIFT single-cell separation systems can achieve efficient and precise isolation and reception of single cells.

### 3.3 High-speed camera capture of sorting process

Figure 4 illustrates the ejection and reception process of *E. coli* under the influence of Gaussian, vortex, and flat-top beams, captured by a high-speed camera. From the ejection of cell from the aluminum film to its landing on the receiving chip, the entire process takes 250 μs, with a distance of 170 μm between the aluminum film and the receiving chip. Through high-speed camera recording, we observe the impact of different beam modes on single-cell sorting clearly. GS-LIFT ejection generates a significant offset, while OV-LIFT ejection produces a smaller offset, and FT-LIFT ejection exhibits almost no offset, achieving highly precise sorting and reception. These results provide visual support for the performance differences among various LIFT systems, further confirm the exceptional capabilities of flat-top and vortex beams sorting systems in achieving high-precision sorting and reception.

### 3.4 Single-cell viability and cultivation capability

**Figure 6:**
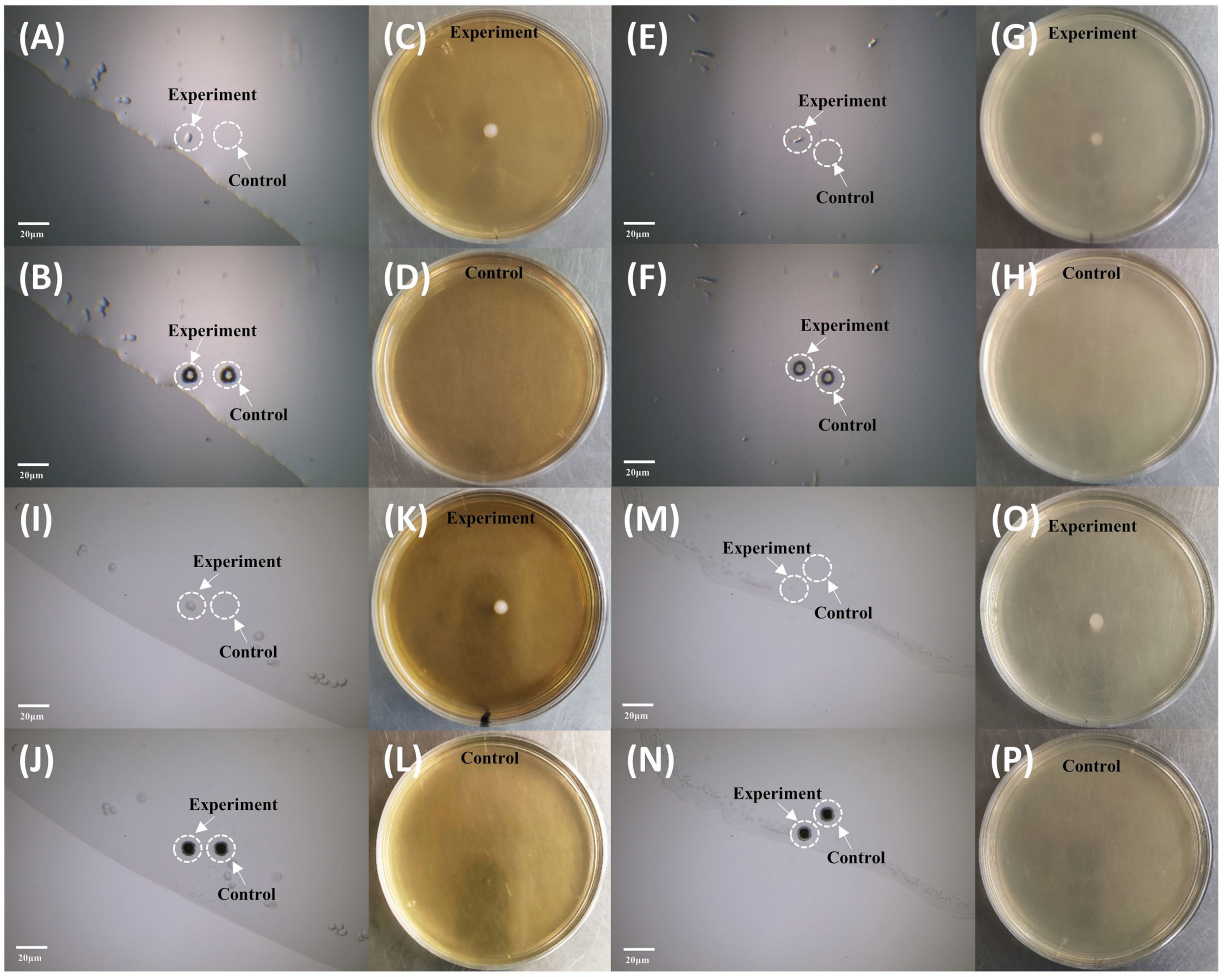
Sorting and cultivation of single cells by vortex and flat-top beams. **(A–D)** Sorting and cultivation of individual *S. cerevisiae* by vortex beam. **(A)** Microscopy imaging before sorting. **(B)** Microscopy imaging after sorting. **(C)** Cultivation of the sorted cell using vortex beam. **(D)** Control group without colony growth. **(E–H)** Sorting and cultivation of individual *E. coli* by vortex beam. **(E)** Microscopy imaging before sorting. **(F)** Microscopy imaging after sorting. **(G)** Cultivation of the sorted cell using vortex beam. **(H)** Control group without colony growth. **(I–L)** Sorting and cultivation of individual *S. cerevisiae* by flat-top beam. **(I)** Microscopy imaging before sorting. **(J)** Microscopy imaging after sorting. **(K)** Cultivation of the sorted cell using vortex beam. **(L)** Control group without colony growth. **(M–P)** Sorting and cultivation of individual *E. coli* by flat-top beam. **(M)** Microscopy imaging before sorting. **(N)** Microscopy imaging after sorting. **(O)** Cultivation of the sorted cell using vortex beam. **(P)** Control group without colony growth. The bar represents 20 μm.

We employed *S. cerevisiae* and *E. coli* as models for cell sorting and cultivation. First, we ejected the blank area near the target cell as control group, and then the target cell was ejected. As shown in Figure 6, the single-cell ejected vortex and flat-top beams could form individual colonies on the corresponding agar plates, and for the control group, in which ejecting the area near the target, no colonies grow. The results confirms that the system can precisely eject cells at single cell level and the sorted single cells could be cultivated further without harm.

### 3.5 Quantitative analysis of viability and cultivation capability

**Figure 7:**
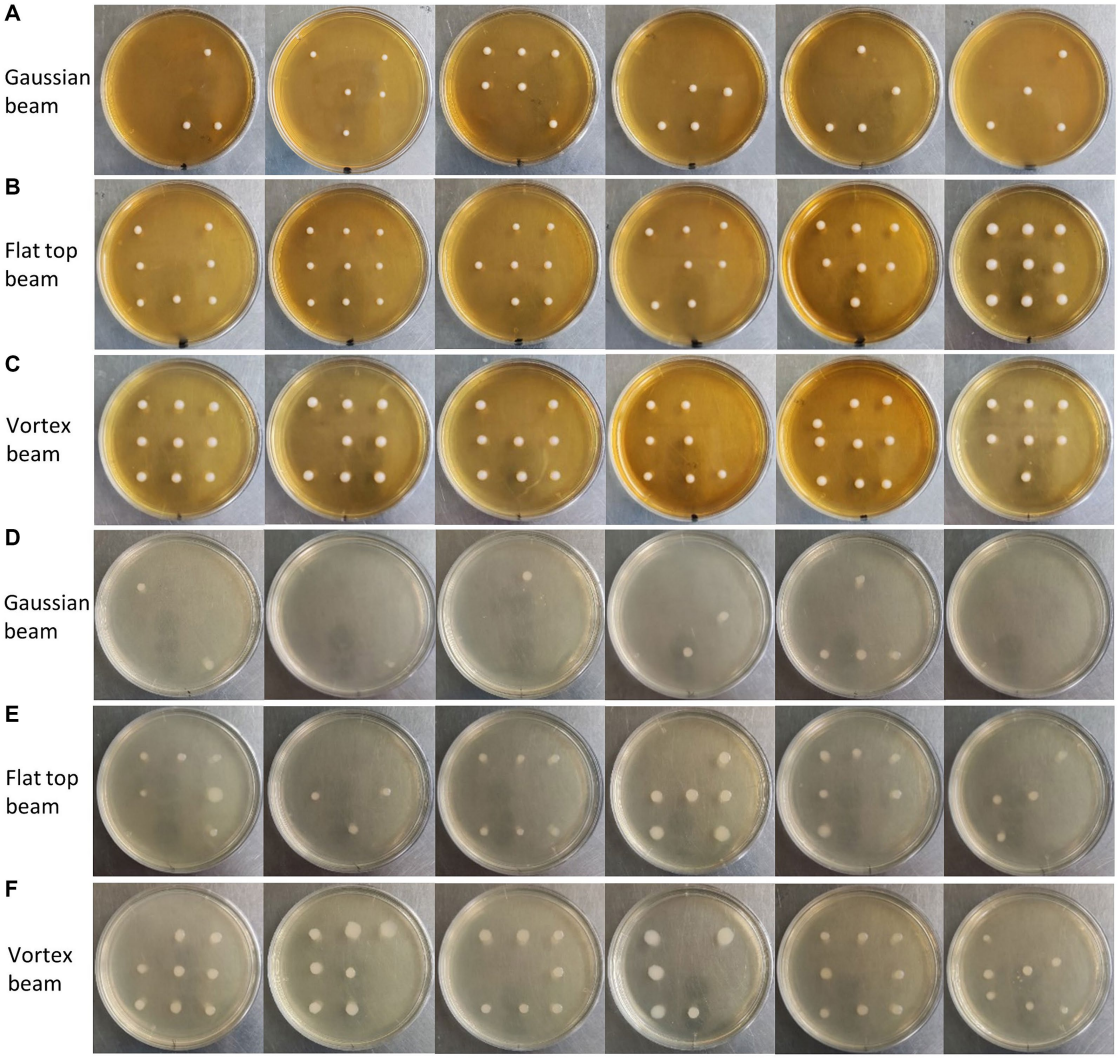
**(A)** Cultivation of single *S. cerevisiae* cells ejected by Gaussian beam into predetermined areas of a petri dish. **(B)** Cultivation of single *S. cerevisiae* cells ejected by flat-top beam into predetermined areas of a petri dish. **(C)** Cultivation of single *S. cerevisiae* cells ejected by vortex beam into predetermined areas of a petri dish. **(D)** Cultivation of single *E. coli* cells ejected by Gaussian beam into predetermined areas of a petri dish. **(E)** Cultivation of single *E. coli* cells ejected by flat-top beam into predetermined areas of a petri dish. **(F)** Cultivation of single *E. coli* cells ejected by vortex beam into predetermined areas of a petri dish.

In Figure 7, we present the single-cell cultivation results of ejecting *S. cerevisiae* and *E. coli* at nine predetermined receiving positions by gaussian, vortex, and flat-top beams, respectively. Six independent repetitions in each experiment were carried to ensure the reliability of the results. For *S. cerevisiae* cells, the average recovery rate was 48% for Gaussian beam ejection, 85% for flat-top beam ejection, and 89% for vortex beam ejection. In the case of *E. coli* cells, the average recovery rate was 19% for Gaussian beam ejection, 57% for flat-top beam ejection, and 78% for vortex beam ejection. This cultivation results demonstrates a significant improvement in recovery rates of *S. cerevisiae* and *E. coli* cells ejected by vortex and flat-top beams compared to traditional gaussian beam.

## 4 Discussion

In this study, we demonstrated a novel approach combining LIFT with vortex and flat-top beam, providing an effective solution for the precise sorting and retrieval of single cells. In comparison to traditional gaussian beam LIFT, our flat-top beam sorting system achieved highly accurate reception within a 50 μm range at a reception distance of 500 mm, presenting a significant advantage. Additionally, the vortex beam sorting exhibited outstanding performance in reducing single-cell damage, with *S. cerevisiae* and *E. coli* survival rates increased to 89 and 78%, respectively.

Despite achieving notable outcomes, there are areas that merit further improvement and exploration. Firstly, we plan to investigate the use of vortex and flat-top beams slides as alternatives to SLM for easier integration into commercial microscopes, enhancing operability and practicality. Secondly, we will delve into the thermodynamic models involved in the sorting process to gain a more comprehensive understanding of the mechanisms behind precise reception and damage reduction. Thirdly, a systematic study is needed to understand the impact of different modes of beams, laser energy density, and sample size on the accuracy and damage of ejected single cells, aiming to improve applicability and stability. Lastly, we plan to integrate femtosecond laser with beam shaping for single-cell sorting, where the femtosecond laser thermal effect is negligible to minimize the damage received by the cells.

In summary, our research provides substantial support for the advancement of single-cell sorting technologies, opening new possibilities for exploration in microbiology and related fields. The successful application of this innovative method will equip researchers in relevant fields with new tools and perspectives, propelling in-depth exploration in microbiology, cell biology, and other related disciplines.

## Data availability statement

The original contributions presented in the study are included in the article/Supplementary material, further inquiries can be directed to the corresponding authors.

## Author contributions

FC: Data curation, Formal analysis, Investigation, Writing – original draft, Writing – review & editing. KL: Conceptualization, Resources, Writing – review & editing. LS: Formal analysis, Writing – review & editing. YW: Conceptualization, Writing – review & editing. XT: Investigation, Writing – review & editing. PL: Methodology, Writing – review & editing. BL: Conceptualization, Methodology, Supervision, Writing – review & editing.
